# A cross-species socio-emotional behaviour development revealed by a multivariate analysis

**DOI:** 10.1038/srep02630

**Published:** 2013-09-11

**Authors:** Mamiko Koshiba, Aya Senoo, Koki Mimura, Yuka Shirakawa, Genta Karino, Saya Obara, Shinpei Ozawa, Hitomi Sekihara, Yuta Fukushima, Toyotoshi Ueda, Hirohisa Kishino, Toshihisa Tanaka, Hidetoshi Ishibashi, Hideo Yamanouchi, Kunio Yui, Shun Nakamura

**Affiliations:** 1 Tokyo University of Agriculture and Technology, Tokyo, Japan; 2 National Institute of Neuroscience, NCNP, Tokyo, Japan; 3 Meisei University, Tokyo, Japan; 4 University of Tokyo, Tokyo, Japan; 5 Saitama Medical University, Saitama, Japan; 6 Ashiya University, Kobe, Japan

## Abstract

Recent progress in affective neuroscience and social neurobiology has been propelled by neuro-imaging technology and epigenetic approach in neurobiology of animal behaviour. However, quantitative measurements of socio-emotional development remains lacking, though sensory-motor development has been extensively studied in terms of digitised imaging analysis. Here, we developed a method for socio-emotional behaviour measurement that is based on the video recordings under well-defined social context using animal models with variously social sensory interaction during development. The behaviour features digitized from the video recordings were visualised in a multivariate statistic space using principal component analysis. The clustering of the behaviour parameters suggested the existence of species- and stage-specific as well as cross-species behaviour modules. These modules were used to characterise the behaviour of children with or without autism spectrum disorders (ASDs). We found that socio-emotional behaviour is highly dependent on social context and the cross-species behaviour modules may predict neurobiological basis of ASDs.

Difficulty in social communication is a major symptom of people with autism spectral disorders (ASDs)^1^. The development of social communication is a complex process^2^,^3^,^4^ including sensory-motor, limbic, and cognitive system maturation and we are still uncertain of neurobiological basis of the symptom irrespective of great progress in neuroimaging^5^,^6^,^7^,^8^,^9^ and epigenetic and molecular study of social neurobiology^10^,^11^,^12^,^13^,^14^,^15^,^16^,^17^,^18^,^19^,^20^. The comparative behavioural study has contributed to improve our concept in affective neuroscience and understanding of cross species neuronal basis of socio-emotional development^10^,^21^,^22^,^23^,^24^,^25^ as briefly described below. A human infant is born with biased “preference” toward con-specific facial features. The study of domestic chick (*Gallus gallus domesticus*) provided clear evidence that facial preference developed through two acquisition steps, predisposition and imprinting^26^,^27^,^28^,^29^. The predisposition is released without any sensory experience of con-specific features, but required non-specific motor activity^24^,^28^. The imprinting stabilizes the predisposition, thus guides sensory attention toward con-specific individuals^30^. The sensory “preference” bias is not only for facial cognition^31^ but also includes cognition in vocalization^32^,^33^, olfaction^34^, self-propelled movement^35^, and biological motion^36^,^37^. Motion perception becomes the bases of mimicking other's behaviour and understanding other's goal-directed behaviour, eventually leading to acquisition of theory of mind^38^,^39^,^40^,^41^.

Another animal model, the common marmoset (*Callithrix jacchus*) has several merits in neurobiological study of socio-emotional development. The marmoset recognizes biological motion (female only among adults)^42^ and is a monogamous species. A pair, as well as young family members, cooperates in raising their offspring together. Furthermore, the marmoset exhibits altruistic behaviour which is a rare among primate species^43^,^44^. These social features make this animal attractive as a model of human social relationship^45^,^46^,^47^,^48^.

In a parallel and interactive manner with sensory-motor system, the development of limbic system confers emotional valence-based learning of behaviour and works together with cognitive processing to represent the expectation of reward and reward prediction error of behaviour choice as a form of belief under uncertainty condition^40^,^49^,^50^. Interestingly, persons with ASD often exhibit hyperactivity or difficulties in sensory-motor information processing^1^. A few examples are fearful face recognition^21^,^23^, restricted visual^51^,^52^ and acoustic attention^1^, tactile sensation^53^, and proprioceptive sensation^39^. Thus, it is important to understand sensory-motor information processing in relation to social communication development.

In this study, we used two animal models, the domestic chick and the common marmoset and compared the social communication behaviour with that of ASD in the light of species specific and cross-species features. The chick was reared under various social sensory deprivation conditions and the development of social communication behaviour was video-recorded and statistically analyzed^54^,^55^,^56^,^57^. The marmoset was reared in three different sibling conditions, two siblings with their parents, single sibling with their parents, and single sibling reared by human care givers^46^,^47^,^48^. The focus in this study was the development of peer-sociality in two animal models. We found cross-species affective behaviours uniquely correlating each other in a statistic space using principal components analysis. The cross-species correlation was also common in social behaviour of children with or without ASD and the difference of their behaviour was characterized by a combination of behaviour modules extracted from socio-emotional behaviour of animal models which were in several developmental stages and had experienced variously social sensory interaction during development. These results may improve the understanding of the core symptom of child with ASD and to develop sensory, motor, emotional, and cognitive intervention for improving their quality of life.

## Results

### Differential social behaviour between high-functioning autistic children and their non-ASD siblings revealed by multivariate behaviour analysis

**Figure 1:**
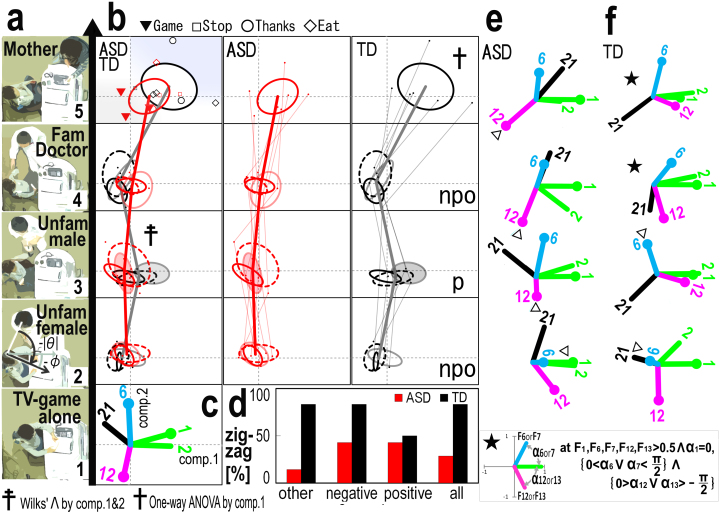
Feature extraction and visualisation of ASD or TD children's behaviour. (a) Top view paintings of the five contexts (1, TV-game alone; 2, an unfamiliar female; 3, an unfamiliar male; 4, a familiar doctor for ASD children and 5, child's mother). All images were originally produced by the authors. (b) The behavioural average plots and distribution ellipses in a PCA plane. A single subject's behaviour was averaged for each context and is expressed as a dot (ASD red, TD black). The trajectory of one subject over contexts is shown by a thin connected line. In contexts two to four, the sub-contexts were set according to the subjects' verbal responses as either positive (p, solid), negative (n, dashed), or other (o, tint) affection. In context five, the subjects played a game (Game), stopped the game (Stop), ate (Eat) and thanked their mothers (Thank); the behavioural type is illustrated using specified marks. The TD children's Thanks behaviour is emphasised using a grey colour region, and the ASD children's Game behaviour is noted with a red triangular region. The cross mark indicates statistically significant differences between TD children's behaviour in context five from all of the other contexts, except for contexts three-n and three-o. The behaviour in the sub-context (o) in context three significantly differed between ASD and TD children (double cross). (c) Factor loading vectors in the common PCA plane were constructed with all of the data, except for age. The vector numbers corresponded with that shown in Table 1. (d) Comparison of sub-context trajectory between ASD and TD. The zig-zag index was the number of the ASD or TD participants who exhibited zig-zag nature out of each participant and expressed as percentage. (e,f) Factor loading vectors of ASD or TD in each context. The angular configuration of the vectors suggests the affiliate state of children and a typical pattern was presented in the right bottom (see text). Atypical location of behaviour parameters was marked by open triangle.

First, a social meeting test was conducted with each participant at a clinic interview room (Figure 1). The participants included seven children aged 8 to 15 years who were diagnosed with high-functioning autism (ASD) according to both the DSM-IV-TR and SRS (Method section) and six typically developing (TD) siblings who have never been diagnosed as ASD or other developmental disorders of similar age-ranges living together in the family. The experimenters (stimulators) (four social contexts, see Method section) asked the participants questions, and the affection of the questions was subjectively categorized as positive (p), negative (n) or other (o) by the experimenters (three sub-context, see also Figures S5 and S6). For example, positive question asked was; “What was your most pleasant experience recently?” As negative one asked was; “Did you have a difficult time at school today?” As neutral question asked was; “Could you give your name please?” The responding behaviours of each participant at five serial contexts (Figure 1a) were video recorded.

From the still video image per sec, the coordinates (x, y) of the participant's and a stimulator's head centre and nose were extracted, and the parameters were listed in Table 1 (Methods section). A principal components analysis (PCA) was conducted based on a correlation matrix of behavioural parameters from all of the participants (Figure 1b, c). The PCA values were plotted in the first and second component plane, and the distribution of the plots for the ASD or TD children was shown separately using a variance ellipse and superimposed on one another (Figure 1b). The shift of the ellipse centre (the average of PCA score) along the first component, particularly in contexts three and five, which suggested that ASD children significantly suppressed head motion (head-central velocity [1] and head-azimuth velocity [2]) and took frequent facial orientations to the stimulator in the sub-context (three-o) within context three (unfamiliar male) when compared to the TD children (see the zigzag ratio in Figure 1d). In the three-o sub-context, the unfamiliar male acted in an impolite manner to ask the participant “Is Dr. Tanaka here?” The PCA values of TD children in context five (mother) shifted to the first quadrant and were significantly different from the values in other contexts (cross mark in Figure 1b), except in the three-o sub-context. The TD scores in context five shifted towards positive along the second component, more so than those of ASD. This result suggests an increase in social affinity (positively correlated with ‘sy-close’ factor loading vector [F6] and negatively correlated with sp-close [F12], as shown in Figure 1c and the Methods section). The rational of the emotional valence of sy-close and sp-close behaviours will be described below.

To compare every behavioural pattern that was exhibited in each context, we examined the PCA planes, focusing on the planar regions in which the length of the factor loading vector of the head-central velocity (F1) was longer than 0.5 (coefficient of correlation from 0 to 1.0), and both the sy-close (F6) and sp-close (F12), given that F1 was aligned to the first component axis (Figure 1e, f). The factor loading vectors equal to |d(phi)/dt| (F2) and -|theta| (head-toward-other preference) (F21) were also represented as significantly contributing factors in the plane. Referring to a hypothesised affective structure, which was based on chick data described in detail later (Figures S1g and S4f), a typical vector correlation structure was noted with an asterisk (TD in contexts 4 and 5), and an atypical vector was noted with an open triangle (Figure 1e, f). In the typical correlation, three factor loading vectors (F1, F6, and F12) configured the defined angularity (the right bottom of Figure 1). In context four (the patient's doctor), head-toward-other preference (F21, correlated with visual attention) highly correlated with sy-close (F6) in ASD, on the while F21 correlated with sp-close (F12) in TD, suggesting that ASD and TD differently use visual attention in the synchronised approach (sy-close, F6).

### Behaviour features in animal models having restricted sensory communication experiences

To further understand the neural basis of the correlation structure of the behavioural parameters in ASD and TD, we needed an animal model whose social behavioural development was similarly analysed (Table 1) to extract a common behavioural feature. Here, we introduced two animal models, the domestic chicks and the common marmoset.

### Chick model

In the chick model, we prepared two basic experimental groups, socially isolated (Iso) and grouped (Grp) chicks. In between them, variously sensory social deprivation groups were prepared as shown in Figure S1. The chicks were reared under each condition for 2 weeks and tested for their social communication behaviour at the last day. We set up three social contexts for the behaviour test, context one as isolation, context two as acoustic only cue (v − a+), and context three as visual and acoustic cues (v + a+). Note that we used different notation for the sensory social deprivation condition and the social context of the behaviour test, that is, a capital letter like V- denotes the deprivation under the rearing condition, on the while a small letter v- denotes the behaviour context. The behaviour parameters listed in Table 1 were averaged during each context, and a PCA was carried out using all of the data collected during the three contexts. In the case of the grouped chicks, the context three (v + a+) ellipse appears in the first quadrant, indicating that active (F1) and positive (F7) behaviour parameters increased over the period of the contextual shift (Figure S1d, FGrp and UGrp). In contrast, the isolated chicks remained immobile throughout each context (Figure S1d). Over all features of the context-dependent ellipse shift was illustrated as the similarity percentage with the grouped and isolated chicks, respectively in Figure S1e. This similarity pattern was further analyzed by a clustering structure of factor loading vectors (Figure S1f). The clustering patterns of the factor loading vectors distinguished FGrp and UGrp (V + A + T+) from other social sensory deprivation chicks. The clustering pattern of grouped chicks was illustrated using the selected behaviour parameters (Figure S1g). Comparing this typical behaviour feature with that of the deprived chicks, the atypical clustering of behaviour parameters was picked up as shown in Figure S1f (open triangle).

### Marmoset model

Next, we analysed long-term behaviour development in common marmosets that were raised under three different social conditions: 1) P2, raised as twins by their own parental caregiver; 2) P1, raised alone by its own parental caregiver; and 3) H1, raised alone by a human parental caregiver (Figure S3a). Behavioural development was examined by a series of four meeting contexts (Figure S3d). With the P2 marmoset, we used two different meeting conditions (i.e., one to an unfamiliar non-sibling [un] and another to a familiar sibling [fs]). The results were classified into three developmental stages, I (P30-60d), II (P80-100d, weaning period), and III (P101-130d). All of the data, including 15 distinct call types over each of the developmental stages (Figure S2) were combined in a correlation matrix, analysed via PCA and plotted as PCA scores. First, we compared the behaviour of four groups (P2 included two sub-groups) with the combined data from three developmental stages. The behavioural features of each rearing group were presented as variance ellipses (Figure S3b). The ellipse of each context was superimposed on each other. P2un and P2fs behaviour did not change appreciably over each context; which contrasted with the grouped chick behaviour. On the while, the context specific behaviour was clear in P1 and H1 (Figures S3b and S3d). P1 differed in three contexts and H1 in the (v + a + o+) context. The P1 and H1 behaviour was different from P2un and P2fs in high frequency of t-call which is emitted to affiliated animals (Figure S3C and Figure S2), suggesting immaturity or different usage of vocalization compared with P2un and P2fs. H1 moved actively compared with P1 and P2 and its ellipse overlapped more with that of P2.

Next, we compared the stage specific behaviour (stages I, II, and III) between four groups. The same data set used in Figure S3 was separately analyzed by PCA and the stage specific variance ellipse was illustrated in 3D time and space (Figure S4c) (235 samples). For comparison, P2un developmental trajectory was super-imposed on P2fs, P1, and H1 trajectories. P2un and P2fs seemed quite similar, while P1 differed at stage II and H1 at stage I. This point was visualized by superimposing four group trajectories in a 3D time and space (Figure S4d). Furthermore, an analysis using factor loading vectors at each developmental stage revealed qualitative and subtle differences among P2un, P2fs, P1 and H1 (Figure S4e). In particular, P2fs and P2un exhibited similar angularity configuration of F1 (head centre velocity), and two approach parameters, sy-close (F6, synchronised approach) and sp-close (F12, spontaneous approach) beyond stage II. H1did not exhibit this pattern and P1 weakly showed this pattern at stage III. These results suggest that P1 and H1 might exhibit learning deficits related to appropriate call usage and approaching behaviour in a social context. Interestingly, P1 could potentially learn these social skills from their parents. However, the similar deficits in P1 and H1 suggest that the skills may be better acquired through interactions between siblings apart from parental instruction presumably because of stage-specific behaviour phenotype and learning ability itself. More statistical treatment of the result will be described below.

### Cross-species affective state revealed by correlation structure of social interaction parameters

Finally, we re-evaluated the similarities and differences of behavioural pattern over species using PCA with five common behaviour parameters from chick, marmoset, and human (Table 2, and Figures S5 and S6). We had eight chick groups (grouped, socially isolated, and variously sensory social deprivation groups, Figure S1), four marmoset groups (P2un, P2fs, P1, and H1), and ten human context groups each for ASD and TD (sub-context positive, negative, and other in context 2, 3, and 4 and context 5, Figure 1). The correlation of the PCA score in each group (total 22 groups) was computed and we have a 22 × 22 correlation matrix expressed as p-value of Wilks' lambda distribution (Figure S6, from different to similar in between 0 < p < 1). First, we closely examined the feature appeared in the sub-context other in context 2 (unfamiliar female), context 3 (unfamiliar male), and context 4 (the patient's doctor) (Table 2 and Figure S5) since the difference of behaviour between ASD and TD was most salient in the sub-context other (Figure 1d).

We tried to understand human behaviour as a combination of animal behaviour features (Figure S5). In context 2, ASD and TD seemed very similar, while ASD and TD were quite different in context 3 and 4. In context 3, ASD behaviour showed similarity with P2fs and P2un. In context 4, ASD behaviour was similar to H1 behaviour, while TD behaviour to P2fs and P2un behaviour. We expanded this type analysis to include chick behaviour (Figure S5 and Table 2). From the extensive matrix score (Figure S5), there is a p-value greater than 0.5. We arranged them in a small matrix focusing on the other sub-context. In context 2, ASD and TD behaviour were quite similar even with chick data, suggesting that both ASD and TD were not active and did not take a particular type of behaviour. In context 3, TD has no score in chick behaviour and ASD behaviour was similar to that of T, A, and Aart only chicks. One possible explanation on why TD has no score in chick behaviour may be that TD took a spontaneous approach to establish a communication with the unfamiliar man. On the while, ASD was merely passively watching the unfamiliar man. In the PCA feature plane (Figure S2a), the ASD behaviour feature was overlapping with that of high frequency of distress call emitted by T, A, Aart chicks, which is consistent with above notion. In context 4, ASD and TD behaved just oppositely, presumably because the doctor was not familiar to TD children. ASD and TD behaviour were context-dependent and the animal behaviour having different social rearing background was useful to characterize the ASD and TD behaviour. More extensive statistic comparison will be described in the next section.

## Discussion

We explored the evaluation of affective states based on digitized behaviour under a particular social context. Psychologists and psychiatrists rely on their diagnoses by the behaviour analysis or intuition of their subjects. This depends on their experience and cultural background. In this study we used video-recorded data of social meeting, extracted digitized parameters, and explored the statistical correlation structure using principal components analysis. In animal models, we can control the social rearing conditions and obtain basic knowledge of individual social interaction history. Furthermore, a series of social meeting contexts can be experimentally designed so as to extract sensory-cues dependent on the modulation of behaviours.

In this study, three methods were used to characterise the behaviour features: first, a transitional pattern analysis of an averaged group data over social meeting contexts (Figure 1) and second, a clustering analysis of all groups' data as factor loading vectors' radiation pattern and an allocation of a variance ellipse in the vector space (Figure S5). Lastly, we visualized a developmental trajectory of marmoset in 3D time and space by PCA. This showed atypical behaviour class as a discrete plot set in the space (Figure S4).

Using common behaviour parameters among human and animal models, we ran a PCA and found a factor loading vector plane which could explain the feature of ASD and TD children behaviour based on statistical similarity with animal behaviours having specified social sensory deprivation history (Table 2 and Figures S5, S6). The correlation of the PCA score in each group (total 22 groups) was computed and we have a 22 × 22 correlation matrix (Figure S6). Taking p = 0.5 as a threshold value, we found a behaviour mapping of ASD and TD children in relation to behaviour modules expressed by animals. As shown in Figure S5 and Table 2, the marmoset behaviour was classified in two categories, **active and sp-close** in H1 and P1 (H1/P1), **not active and not affective** in P2fs and P2un (P2). The chick behaviour was also classified into four categories, **affiliate** in VATfam, VATunfam (VAT), and VA (here we omit + mark from V + A + T+ etc. just for simplification), **not active** in V and A, **alert** in Aart, and **immobile/anxious** in V and I (Figures S1 and S6). This categorization is consistent with the clustering pattern of the factor loading vectors in Figure S5a and these behaviour types may reflect a kind of behaviour module which could be cross-species, species-specific, and developmentally stage-specific.

We examined the ASD and TD behaviour types in a specified social context using these animal modules (categorization according to **module class**, Figure S6). Interestingly, in context 5 (children's mother), ASD and TD behaved similarly each other and the module class is marmoset H1/P1. ASD module class is also chick VAT/VA, suggesting a straightforward expression of their affiliation to their mother, not like TD. In contrast, in context 3(unfamiliar male), ASD behaviour class is marmoset P2 and chick V/A as well as Aart irrespective of sub-context (though we found not much correlation with Aart in negative sub-context). On the other hand, TD behaviour class is H1/P1 in sub-context other and negative and showed sp-close behaviour, suggesting their intension of establishing a communication with the man. In context 4 (the patient's doctor), ASD and TD behaviour types are P2 and chick V/A, except ASD in sub-context other where ASD types were H1/P1 and chick VAT/VA as well as Aart, suggesting ASD were active and showed sp-close to their doctor. TD was not active and may be in alert (like chick Aart type) with the unfaimiliar doctor for them. In context 2(unfamiliar female), both of ASD and TD behaviour were very similar in all sub-context as described in Figure S5 and Table 2. Exception was ASD behaviour in sub-context negative, where ASD was more inactive and showed a kind of feature-less behaviour. We could not find any counterpart of chick V and I types in the human subjects.

The socio-emotional responses of ASD and TD subjects were highly dependent on the social context. First of all, familiarity largely affected their response. Both of them showed affiliated behaviour to their mother, but ASD and TD subjects differently expressed their affiliation. Secondly, the emotional valence expressed by the stimulator modified the children's response. ASD and TD behaved similarly to positive valence, but differently behaved to neutral and negative ones depending on the context.

This study has a limitation of sample size and we note that the human control group is composed of the unaffected children whose siblings are autistic. On the one hand, this offers a control of experiential factors, since ASD and TD control subjects share the same living environment. On the other hand, unaffected siblings of autistic subjects may share the so called "broader autistic phenotype". Further study could include a control group from the general population. We needed another consideration in sample size in relation to PCA. In general, a problem of PCA on small data set relates with “a curse of dimensionality”^58^ (C. Bishop wrote in his textbook, “Not all intuitions developed in spaces of low dimensionality will generalize to spaces of many dimensions.” In our case, we can differentiate a data class from other classes by chance if we had very larger number of parameters than that of the subjects.). One idea to overcome the challenge of having a small sample size is following. First of all, we have many independent studies of chick social behaviour and published the result of PCA which showed the segregation of two experimental groups, reared as a group and as social isolation conditions^54^,^55^,^56^,^57^. Taking the advantage of robustness of segregation of two group behaviours in a feature space after PCA, we combined all the data of two groups, constituted an authentic space, and embedded a new test data to discriminate its identification in the space. This method successfully predicts the identification of this test data whose total number is 292. In the case of the marmoset, we do not have as many individuals as chicks, though we published a few papers on the marmoset behaviour analysis using PCA^46^,^47^,^48^. Another idea to overcome the challenge of having a small sample size is to introduce a longitudinal study instead of a cross-sectional study. In the result shown in Figures S3 and S4, we prepared three experimental groups, two siblings reared by their parents, one sibling reared by its parents, human reared ones with n = 4 ~ 13 with each group in each of three contexts. Starting from postnatal day 30, we followed social behaviour development until postnatal day 210 and performed more than ten times behaviour test in total. In this situation, we can apply PCA on more than two hundred individual data and express the group behaviour feature as the trajectory in 3D time and space. By this way, we could discriminate behaviours between two siblings, a single sibling, and human reared. As another consideration for “a curse of dimensionality”, we note that behaviour parameters are usually not mutually independent. In fact, several parameters spontaneously clustered together. Thus, we used as many parameters for the first step of behaviour analysis, selected major parameters which heavily contributed in a feature space based on the vector length (more than 0.5 after normalization), and ran the second PCA using the selected parameter, keeping the number of parameters to at least less than half of the sample size.

The correlation analysis by PCA is a statistical model-free procedure. On the other hand, the correlation structure is essentially restricted by the sample set and we have no idea how the goodness of the correlation structure is as a prediction model for a new data set (though an embedding method is available as described above). Thus, we need to develop a model of socio-emotional development in a state space^59^ using behaviour and neurobiological data, which is certainly an important direction in further research.

## Methods

### Human study

#### Participants

Seven children were diagnosed as high functioning autism (ASD, 7 to 15 years [y], mean 10.9 y, SD 2.9 y, five males and two females) based on DSM-IV-TR^60^ with referring to the scores of Social Responsiveness Scales (SRS)^61^, and as the exclusion criteria, were not matched on either axis I or II of the patient version of the Structured Clinical Interview for DSM-IV-TR^60^. Their verbal and performance IQs were confirmed as >80 at baseline as measured by the Wechsler Intelligence Scale^61^. Five volunteers enrolled were typically developing siblings who had never been diagnosed as ASD or other developmental disorders (TD 8 to 13 y, mean 10.2 y, SD 2.7 y, one male and four females) living together under the same parental care as the participants with ASD. The study protocol was approved by the ethics committee of the Sawa Hospital (Osaka, Japan)^61^. The protocol was registered with ClinicalTrials.gov of the NIH (NLM_DES S0002KCR). Written informed consent was obtained from the participants' parents, directly from the participants or both. The participants' mothers also participated in the study as social stimulators under the approved protocol.

#### Meeting test

Before the start of the meeting test, each participant sat at a desk in the centre of the clinical room and played a TV-based videogame as the default context. Four serial contexts followed in which participants were exposed to different social and emotional stimuli. Briefly, an unfamiliar female (a clinical staff), an unfamiliar male (a volunteer graduate from Tokyo Univ. of Agri. & Technol.), the patient's doctor, and the patient's mother entered the room in this order, sat, and talked or asked questions to the child to induce a positive (p), negative (n) or other (o) type of emotional state (see text). The behaviour of the participants was video-recorded and analysed as described below. The emotional valence of each question was subjectively assigned as positive, negative, and other by MK and AS. Behaviour parameters were identified using the still video image per sec as follows:

1. V [/sec], the velocity of the participant's head centre was calculated per second. 2. |d (phi)/dt| [degree/sec], the rotation velocity of the participant's head centre to nose was calculated per second. 3. Sy-, sp-closer [%], the distance (d_n_) between a participant and a stimulator at n [sec] and the relative distance (d_n−1_) between the participant at n − 1 [sec] and the stimulator at n [sec] was calculated. The relative velocity v = d_n_ − d_n−1_ was defined as a relative movement velocity of the participant and standardised (v′). Close behaviour was defined by v′ > 1. Instances when both a participant and a stimulator behaved ‘close’ together and a participant continued ‘close’ behaviour for the following one second under the stimulator earlier was defined as ‘sy-close’. Cases in which ‘close’ behaviour of the participant was earlier or without ‘close’ behaviour of the stimulator were defined as ‘sp-close’. 4. -|Theta| [degree]: Given that the participant's head centre-nose direction equals the direction of the participant's head centre-stimulator head centre, the participant's angular direction was defined as zero and took on negative values when the angle became large (i.e., the participant did not orient to the stimulator).

### Animal study

The experimental animals included the domestic chick (*Gallus gallus domestics,* white leghorn, Maria) and the common marmoset (*Callithrix Jacchus*). The experimental protocols were approved by the Animal Care and Use Committee of the Tokyo University of Agriculture and Technology and National Institute of Neuroscience (Tokyo, Japan). These committees follow the guidelines of Japanese Neuroscience and the Society of Neuroscience in the United States.

#### Chicks

Fertilised eggs of white leghorn chickens (*Gallus gallus domestics,* Maria) were purchased from Tomaru Farm (in Gifu-City) and incubated at 37.7°C until embryonic day 20 (E20). At E21, usually 1 day before hatching, an embryo often starts calling on this day. Thus, at E21, the eggs were moved to either a home cage under grouped conditions (Grp) or separately under one of six types of socially isolated conditions (Iso). The separated condition provided a rearing environment in which a chick was isolated from other individuals by restricting specific sensory cues as follows: **V + A + T−,** chicks were reared in cages separated by transparent and through-hole board, therefore, they could communicate with each other through visual (V+) and auditory (A+) cues but not via tactile cues (T−); **V − A − T+**, a chick interacted with other grouped chicks for 30 minutes per day with opaque patches over both eyes and ears. The eye patch was equipped with an opaque window and the window was open in the home cage to feeding and closed when the meeting. Although M. Konishi used the extirpation of chick cochlea to deprive auditory sensation^62^, our operation was intended for the social deprivation and attenuated auditory sensation.; **V − A + T−**, a chick was reared in a box consisting of opaque walls and an open ceiling, thus being isolated from visual (V−) and tactile sensation (T−) but not deprived of audition of vocalization from other individuals; **V − Aart + T−**, reared similarly to chicks in the V − A + T− condition, but exposed only to the playback of recorded j-calls for 24 h (j-call was pleasant call or food-related call, i.e., chicks emitted this call while pecking foods^33^); **V + A − T−**. a chick was reared in dual transparent acrylic box; **V − A − T−**, a chick was reared in box of opaque walls and the box was further covered by sound absorbing materials (glass wool). Auditory social deprivation was performed by keeping the subject in dual acryl box which was located beyond 2 meter from the next cage under A − V− and A − V+ rearing conditions. The area of the Grp and Iso home cages varied, e.g., 17 cm wide × 20 cm deep × 17 cm high, 20 cm wide × 20 cm deep × 20 cm high, 37 cm wide × 32 cm deep × 33 cm high or 40 cm wide × 50 cm deep × 50 cm high. The cage walls were made of sound absorbing materials to limit call interactions between birds.

#### Marmosets

Marmosets (*Callithrix Jacchus*) of two different colonies from the National Institute of Neuroscience were used. The first colony was borrowed from Dainippon Sumitomo Pharma Co., Ltd., and the second colony was delivered from CLEA Japan Inc. (Tokyo). Individuals were reared in three different conditions: 1) as two peer siblings in the care of their parents (P2), 2) a single individual in the care of the parents (P1), and 3) a single individual in the care of human caregiver (H1) who fed the animal until weaning. The following sample sizes were used for each group of marmosets: P2 (seven females and six males), P1 (two females and two males) and H1 (three females and three males). Each family was kept in a visual isolation cage. Behavioural tests were recorded twice at each stage; at 35–60 days old (P35-60d), P80-100d, and P101-P130d. With P2 animals, test was performed at P131-210 and adult (only for P2un).

#### Behaviour recording

Digital video cameras (SONY, Tokyo, Japan) and microphones were used to record all behaviour during the meeting test. All of the video material was converted into serial JPEG image files and WAVE sound files using TMPGEnc software (Pegasys, Tokyo,Japan). The XY coordinate was quantified using Image J software. Spectrograms of vocalisations were analysed with Syrinx software (Dr. John Burt, University of Washington, http://www.syrinxpc.com, 1^st^ August, 2013). We differentiated three types of chick calls: d-call, j-call and dj-call^33^. Marmoset calls were categorised into three types (Figure S2)^46^: p-call, thought to signify an ‘anxious’ state, e-call, thought to convey being ‘strained’ (egg, highegg, bass, high, higheggbass or the strong alert call ‘gugaga’) and t-call, thought to indicate ‘comfort’ or ‘relaxation’ (trill, peep, short, short-combination, trillphee, twitter, twitterhead, U, tsik, trillphee).

### Principal components analysis (PCA) and statistics

We used PCA based on a correlation matrix to find correlations between behaviour parameters using Microsoft Excel-based software freely distributed by Yasunori Nakano (http://211.13.211.3/soft/winnt/business/se412290.html, 1^st^ August, 2013) and by Kimio Kanda (http://www.vector.co.jp/soft/win95/edu/se203904.html, 1^st^ August, 2013).

Behaviour parameters were extracted from video recordings, as described above. The correlation matrix of behaviour parameters was then computed and used for the PCA. Subject data were plotted on the appropriate PCA plane. The results of multivariate analysis were visualised in 3D-graphs using Origin ver7.5 software (Origin Lab Cooperation). The factor loadings (FL) vector of behaviour parameters were defined as the product of the square root of the eigenvalue (lambda)_i_ and eigenvector v_i_, as 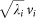 (square root of lambda i multiplied by nu i), indicating the correlation between an eigenvector (principal component) and behaviour parameter. To visualise a group dataset, we used variance ellipses, whose long and short axes were the first and second components of the second PCA completed through a variance-covariance matrix and multiplied by the square root of the eigenvalue using the group data set. The similarity of two group datasets was evaluated by examining the percentage of overlap between the two ellipses. Finally, multivariate analyses were performed using Wilks' lambda distribution via R software to evaluate the statistical significance of the difference between the PCA scores of two group datasets.

## Supplementary Material

Supplementary Information Supplemental Figures S1-S6

## Figures and Tables

**Table 1:** Comparative parameter sets in three animal species. The affective behavioural parameters F1-21 were categorised via clustering analysis after multivariate analysis based on principal components analysis (PCA) (see text). Two locomotive (active and immobile) and two affective (positive and negative) directions were differentiated with colour-coding, which is applied throughout the Figures. The set containing parameters F1, F6/7 and F12/13 were particularly emphasised to discriminate a homologous structure of multi-parametric correlations beyond species differences

F		child	marmoset	chick	parameters
1	active	V	V	V	head-central velocity^26^,^27^,^28^
2		|d*ϕ*/dt|	|d*ϕ*/dt|	|d*ϕ*/dt|	head-azimuth velocity^26^,^27^,^28^
3		–	–	LP-G	closest quadrisection preference^26^
4		–	–	pk-wall	wall-peck frequency^26^
5		–	–	g-move	grouping behavioural marker^26^
6	positive	sy-close	sy-close	–	synchronized approach-to-other frequency^27^,^28^
7		(positive speech)	t-call	j-call	positive-emotional vocalisation^26^
8		–	–	j-call m	j-call morphological number^26^
9		–	–	dj-call	transient (d-j) call frequency^26^
10		–	–	dj-call m	dj-call morphological number^26^
11		–	–	pk-floor	floor-peck frequency (feeding & foraging)^26^
12	negative	sp-close	sp-close	-	spontaneous approach-to-other frequency^27^,^28^
13		(negative speech)	p-call	d-call	negative-emotional vocalisation frequency^26^,^27^,^28^
14		(negative speech)	e-call	(d-call m)	negative-emotional vocalisation frequency^27^,^28^
15		–	shake	–	upper-body shake (alert behaviour)^27^,^28^
16	immobile	–	–	freeze	freezing duration ratio^26^,^27^,^28^
17		–	–	LP-C	central quadrisection preference^26^
18		–	–	LP-E	farthest quadrisection preference^26^
19		–	–	LP-O	other quandrisection preference^26^
20		–	–	pk-self	self-peck frequency^26^
21		−|*θ*|	|*θ*| < 45°	|*ϕ*| < 45°	face to peers^26^

**Table 2:** Human behaviour mapping using behaviour parameters derived from marmoset and chick models. The correlation of ASD and TD behaviours in the specified social context and animal behaviours with variously social sensory deprivation were analysed using five common behaviour parameters (F1, 2, 6 or 7, 12 or 13, and 21 in Table 1 and Figure S5). After PCA, the correlation of the PCA score of each group (total 22) was evaluated by Wilks' lambda (Figure S6). p-value larger than 0.5 was picked up in a 6 × 12 matrix. The sub-context of human behaviour is “other” in context 2, 3, and 4. The marginal p-value close to 0.5 are parenthesized. The left column shows animal models, top 4 for marmoset (Figure S4), bottom 8 for chick (Figure S1). The nomenclature chick social sensory deprivation was same with that depicted in Figure S1 except the omitting + or − mark after V, A, and T and showing + sensation only

condition	2o ASD	2o TD	3o ASD	3o TD	4o ASD	4o TD
H1				(0.46)	0.98	
P1				0.63	0.55	
P2fs	0.85	0.89	0.98			0.74
P2un	(0.47)	0.72	0.98			0.74
VAT fam	(0.49)	0.6			0.65	
VAT unf	0.58	0.63				
VA	0.78	0.61				
T	0.56	0.75	1			0.7
A	0.6	0.75	0.86			0.66
A art			0.52		0.58	0.71
V						
I						
