# Supplementary material for: A cross-species socio-emotional behaviour development revealed by a multivariate analysis
Source: Sci Rep. 2013 Sep 11;3:2630. doi: 10.1038/srep02630 (PMC6505395; doi:10.1038/srep02630)
Supplement: Supplementary Information — Supplemental Figures S1-S6 [file srep02630-s1.pdf]

Supplemental Information

Title:

A cross-species socio-emotional behaviour development revealed by a multivariate analysis

Author lists:

Mamiko Koshiba[1,2,5*], Aya Senoo[1], Koki Mimura[1], Yuka Shirakawa[1], Genta Karino[1], Saya Obara[1], Shinpei Ozawa[1], Hitomi Sekihara[1], Yuta Fukushima[1], Toyotoshi Ueda[3], Hirohisa Kishino[4], Toshihisa Tanaka[1], Hidetoshi Ishibashi[2], Hideo Yamanouchi[5], Kunio Yui[6], Shun Nakamura[1,2]

1. Tokyo University of Agriculture and Technology, Life science and Biotechnology, Tokyo, Japan
2. National Institute of Neuroscience, NCNP, Tokyo, Japan
3. Meisei University, School of science and engineering, Tokyo, Japan
4. University of Tokyo, Tokyo, Japan
5. Saitama Medical University, Saitama, Japan
6. Ashiya University, Kobe, Japan

*Corresponding Author; Mamiko Koshiba, 2-24-16, Naka-cho, Koganei, Tokyo, Japan, Tokyo University of Agriculture and Technology, Tel 81-42-388-7770, Fax 81-42-388-7714, E-mail koshiba@cc.tuat.ac.jp

Supplemental information

Figure S1

Development of affective behaviour under restricted sensory interactions in the domestic chick

(a) The top view of the chick meeting test. **(b)** The meetings in three contexts (isolation, acoustic only cues (v-a+) and visual and acoustic cues (v+a+). The definition of local preference (LP) is shown as (E) Escape (red, farther from peers), (C) Centre (purple), (G) Group (blue, nearest to peers) and (O) Other (grey). **(c)** A schematic presentation of the rearing conditions, grouped together (grouped), socially isolated (Isolated), various sensory-deprivation conditions, visual only cues (V+A-T-), playback of chick calls only cues (V-Aart+T-), acoustic only cues (V-A+T-), tactile only cues (V-A-T+) and visual and acoustic cues without tactile cues (V+A+T-). A Grouped chick met either familiar (FGrp) or unfamiliar (UGrp) peers in a separate meeting test. **(d)** Visualisation of meeting behaviour features in a PCA plane and the shift of distribution ellipses over the contexts are shown (contexts one, two and three, black, grey and blue, respectively). The distributions were significantly different (Wilks' lambda distribution) compared to those of UGrp (asterisk) or isolated (cross) in context three. **(e)** Behaviour similarities in context three between grouped (black square) or isolated (open triangle) expressed as a percentage of overlapping distribution ellipses (Methods section). **(f)** Factor loading vectors expressing the correlation of chick meeting behaviour parameters (Table) in each rearing condition. The black star indicated similar patterns and the open triangle indicated different patterns in relation to a hypothetical affective correlation structure shown in Figure S1g and Figure S4f. (**g**) Factor loading vectors of Figure S1d.

Figure S1

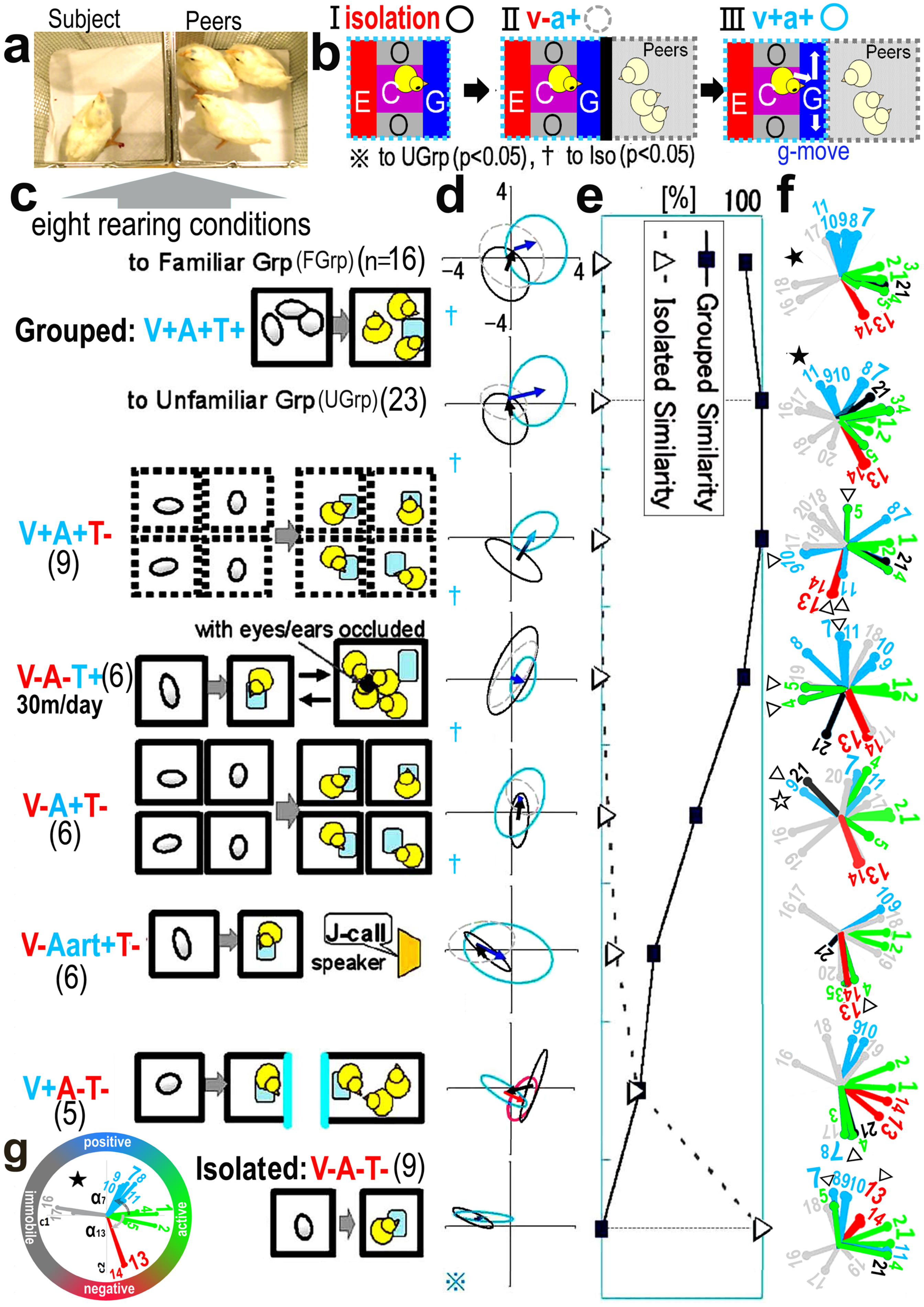
a
Subject
Peers
b
I isolation
II v-a+
III v+a+
E C G
Peers
g-move
※ to UGrp (p<0.05), † to Iso (p<0.05)
c
eight rearing conditions
d
e
f
[%]
100
Grouped Similarity
Isolated Similarity
to Familiar Grp (FGrp) (n=16)
Grouped: V+A+T+
to Unfamiliar Grp (UGrp) (23)
V+A+T-
(9)
with eyes/ears occluded
V-A-T+ (6)
30m/day
V-A+T-
(6)
V-Aart+T-
(6)
J-call
speaker
V+A-T-
(5)
Isolated: V-A-T- (9)
g
positive
negative
immobile
active

Figure S2

Marmoset's call library.

All call data are presented chronologically in each meeting test context. Marmoset rearing conditions (P2, P1 and H1 together with meeting subject familiarity) are demarcated by symbols presented in the box appearing in the upper left. Males and females were distinguished using colour coding. Phee calls (p-call) decreased over developmental stage. T-calls were observed more frequently in context v+a+o+ in P2fs. In contrast, e-calls were emitted in any group meeting with unfamiliar peers.

Figure S2

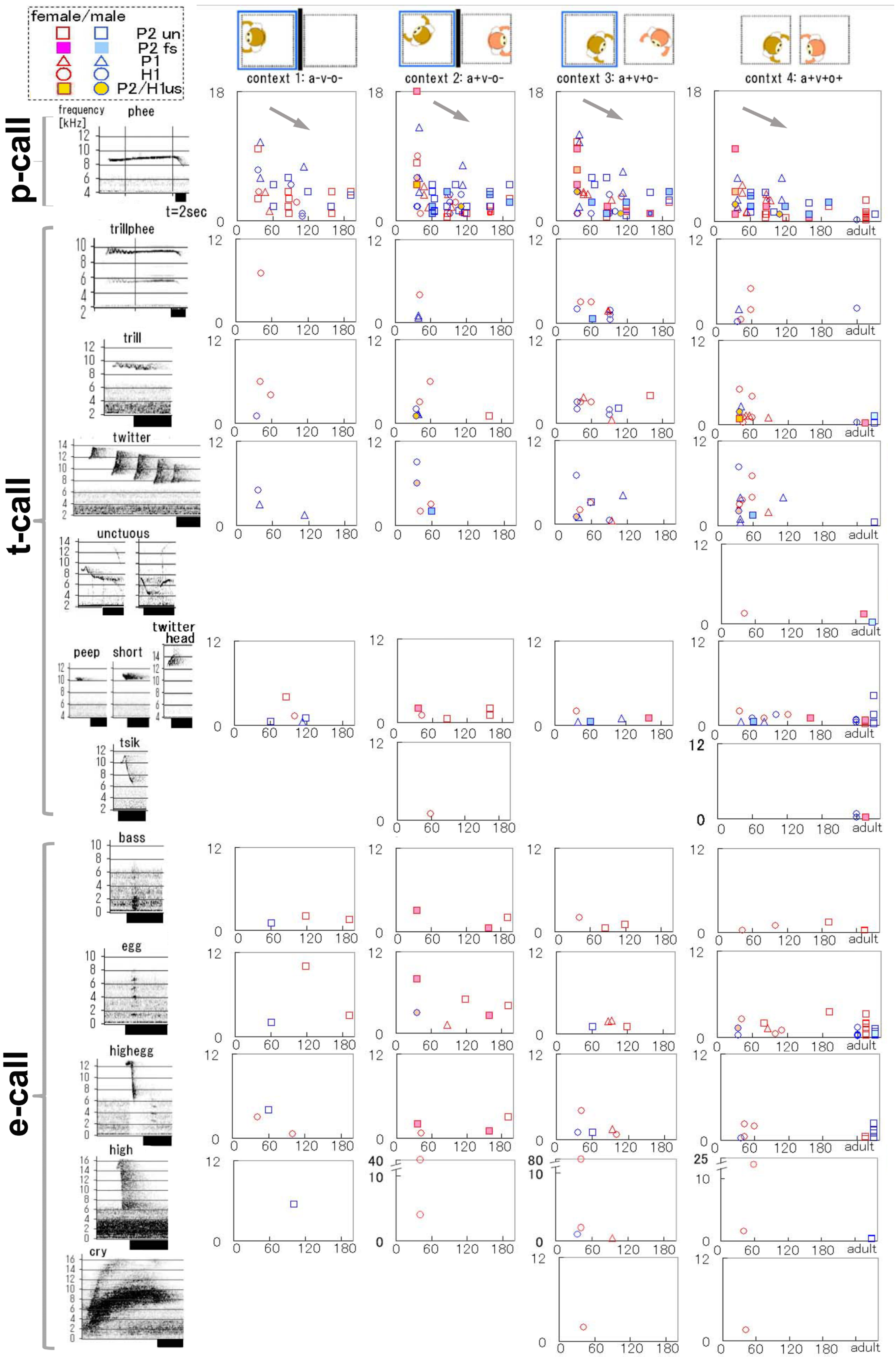

female/male
P2 un
P2 fs
P1
H1
P2/H1us
context 1: a-v-o-
context 2: a+v-o-
context 3: a+v+o-
contxt 4: a+v+o+
frequency [kHz]
phee
t=2sec
p-call
trillphee
trill
twitter
unctuous
peep
short
twitter head
tsik
t-call
bass
egg
highegg
high
cry
e-call
adult

Figure S3

Development of affective behaviour in marmosets experienced various social interactions with peers.

(a) Rearing conditions of marmosets, P2 (two siblings), P1 (without sibling) and H1 (human-reared). The meeting test for P2 was conducted with either an unfamiliar peer (P2un) or a familiar sibling (P2fs). **(b)** Variance ellipse distributions including the data from all of the developmental stages. **(c)** The correlation structure of meeting behaviour vectors in the PCA analysis is shown in (b). **(d)** The similarity analysis for each context in relation to P2un's behavioural features identified by ratios of overlapping areas between variance ellipses. Asterisks indicate significant Wilks' lambda distribution.

*We have produced the cartoon and the corresponding author holds the copyright.

Figure S3

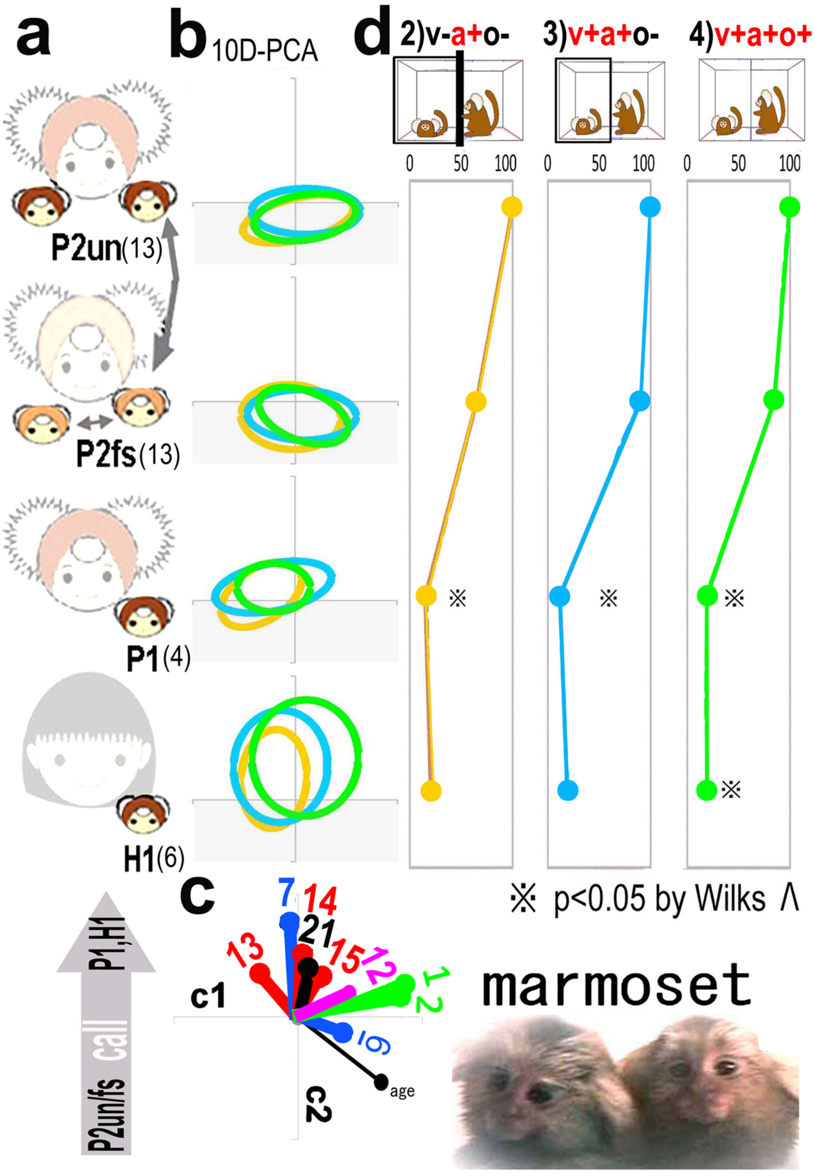

a
b 10D-PCA
d 2)v-a+o-
3)v+a+o-
4)v+a+o+
0 50 100
P2un(13)
P2fs(13)
P1(4)
H1(6)
※
※ p<0.05 by Wilks Λ
c
c1
c2
age
P2un/fs call P1,H1
marmoset

Figure S4

Developmental trajectory in 3D time and space and factor loading vector correlation of marmosets experienced various social sensory interactions.

(a) Developmental trajectory for each developmental stage, (I : P30-60 postnatal day [d], II : P80-100d, III : P101-130). Behaviour data from all contexts were combined and plotted in the PCA plane (first and second components). **(b)** The correlation structure of factor loadings vectors of e-g and i. **(c)** Development of social behaviour of each group. PCA score were plotted in 3d time and space from top, P2un, P2fs, P1, and H1. **(d)** Developmental curve of peer social behaviours in the same 3D time space. Specific regions (red plots circulated by pink dashed hand-writing ellipses) exist, which consist of H1 or P1 plots allowing for some P2fs individuals but no P2un individuals. P2un plots distributed within the line connected between the most outer P2un plots per developmental stage. **(e)** Comparison of correlation structures of factor loading vectors per developmental stage (I, II, and III) with the additional stages P2, P131-210d and adult. The plot of the P2un, P2fs, P1 and H1 animals was aligned in a row. **(f)** A hypothetical correlation structure in social affective behaviour derived from chick data of Figure ure S. 1g. Factor loading vector i (F_i) and the angle (radian) between F_i and the first component were defined ($alpha_i$). A typical affective structure was defined by F1, F6, F7, F12 and F13, and two axes were hypothesised as active-immobile and positive-negative axes.

*We have produced the cartoon and the corresponding author holds the copyright.

Figure S4

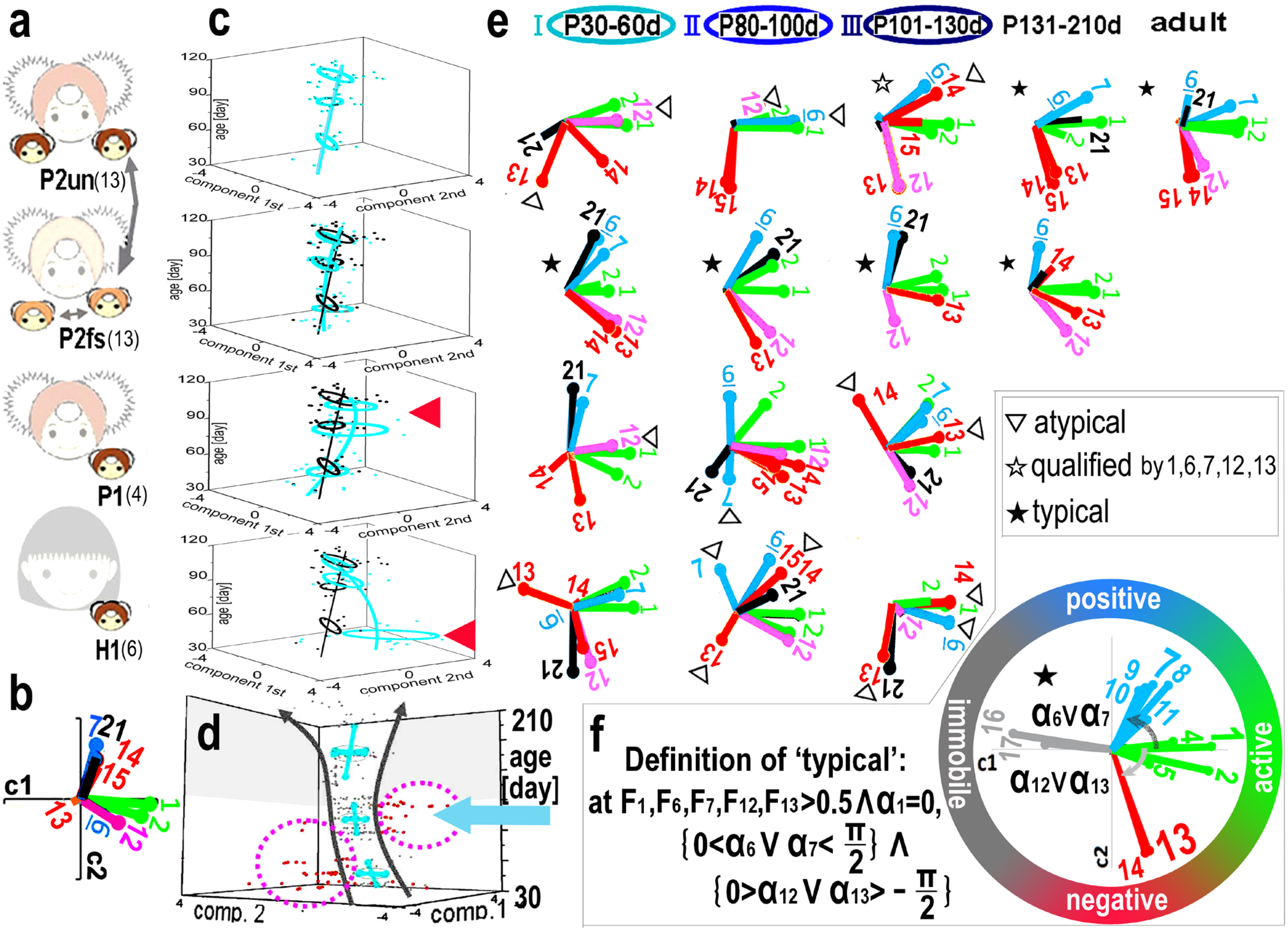
a
P2un(13)
P2fs(13)
P1(4)
H1(6)
b
c1
c2
c
component 1st
component 2nd
age [day]
d
210
age
[day]
30
comp. 2
comp.1
e
Ⅰ P30-60d
Ⅱ P80-100d
Ⅲ P101-130d
P131-210d
adult
▽ atypical
☆ qualified by 1,6,7,12,13
★ typical
f
Definition of 'typical':
at F1,F6,F7,F12,F13>0.5∧α1=0,
{0<α6 V α7< π/2} ∧
{0>α12 V α13> - π/2}
positive
active
negative
immobile
α6Vα7
α12Vα13

Figure S5

Comparison of behaviour patterns across species via PCA with five common behavioural parameters.

a. Factor loadings vector with five parameters, i.e., head-centre velocity (1), head-azimuth velocity (2), sy-close (6) or j-call in chick (7), sp-close (12) or d-call in chick (13) and view-to-peer preference (21) (Table, see factor loadings in Figure ure S. 4). b. The difference between animal and human behaviour compared to ASD (red) or TD (black) at the specified sub-contexts. Each subject's PCA scores using the five common parameters (Figure S3k) were statistically compared using Wilks' lambda distribution, and the p-values are represented in a bar graph as 0<P<1 (different-similar). The variance ellipses are drawn on the PCA plane (the first and second component), ASD (red), TD (black), and animal (grey) and compared between ASD or TD (each sub-context) and marmoset. The overlapping region is illustrated in a dark colour.

Figure S5

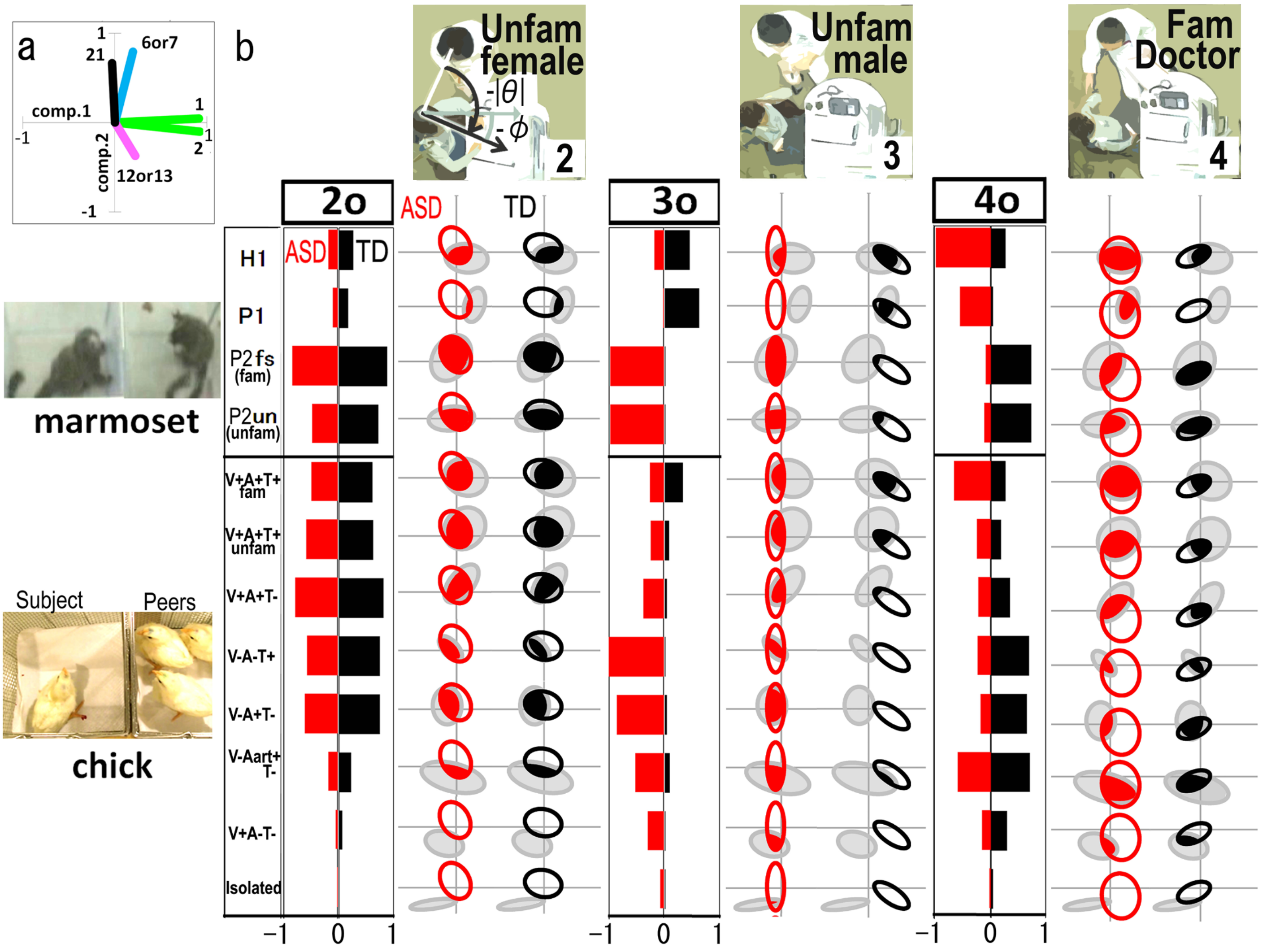

a
1
21
6or7
comp.1
1
-1
1
2
comp.2
12or13
-1
b
Unfam female
-|θ|
-φ
2
Unfam male
3
Fam Doctor
4
2o
ASD
TD
3o
4o
H1
ASD
TD
P1
P2fs (fam)
P2un (unfam)
V+A+T+ fam
V+A+T+ unfam
V+A+T-
V-A-T+
V-A+T-
V-Aart+ T-
V+A-T-
Isolated
marmoset
Subject
Peers
chick
−1
0
1

Figure S6

The extensive comparison of behaviour patterns across species.

Behaviour parameters of the chicks and the marmosets reared in different conditions (total eight groups of chicks and four groups of marmosets as shown in Figure S2 and Figure S3, respectively) were statistically analysed as follows. The parameters were standardised for each species. Chick data were derived from context III in Figure S1b. The marmoset data were used only from stage III (P100-200d). Since the difference between contexts two to four (Figure S3b, S3d), the data of the three meeting contexts were combined. TD and ASD behaviour parameters in 10 sub-contexts (Figure 1) were also standardised. Animal and human data were then combined in a correlation matrix and analysed by PCA. PCA scores from all subjects were compared by Wilks' lambda distribution and a 22 x 22 correlation matrix (p-value) was obtained. The p-value is expressed via colour gradation (Figure S5a) or bar graph (Figure S5b). In Figure S5a, the result of the within-subject comparison is expressed as a black square, and the ASD-TD comparison within the same sub-context is highlighted by a diagonal line and the significant dissimilarity ($p<0.05$) between them was highlighted with white outlined squares.

Figure S6

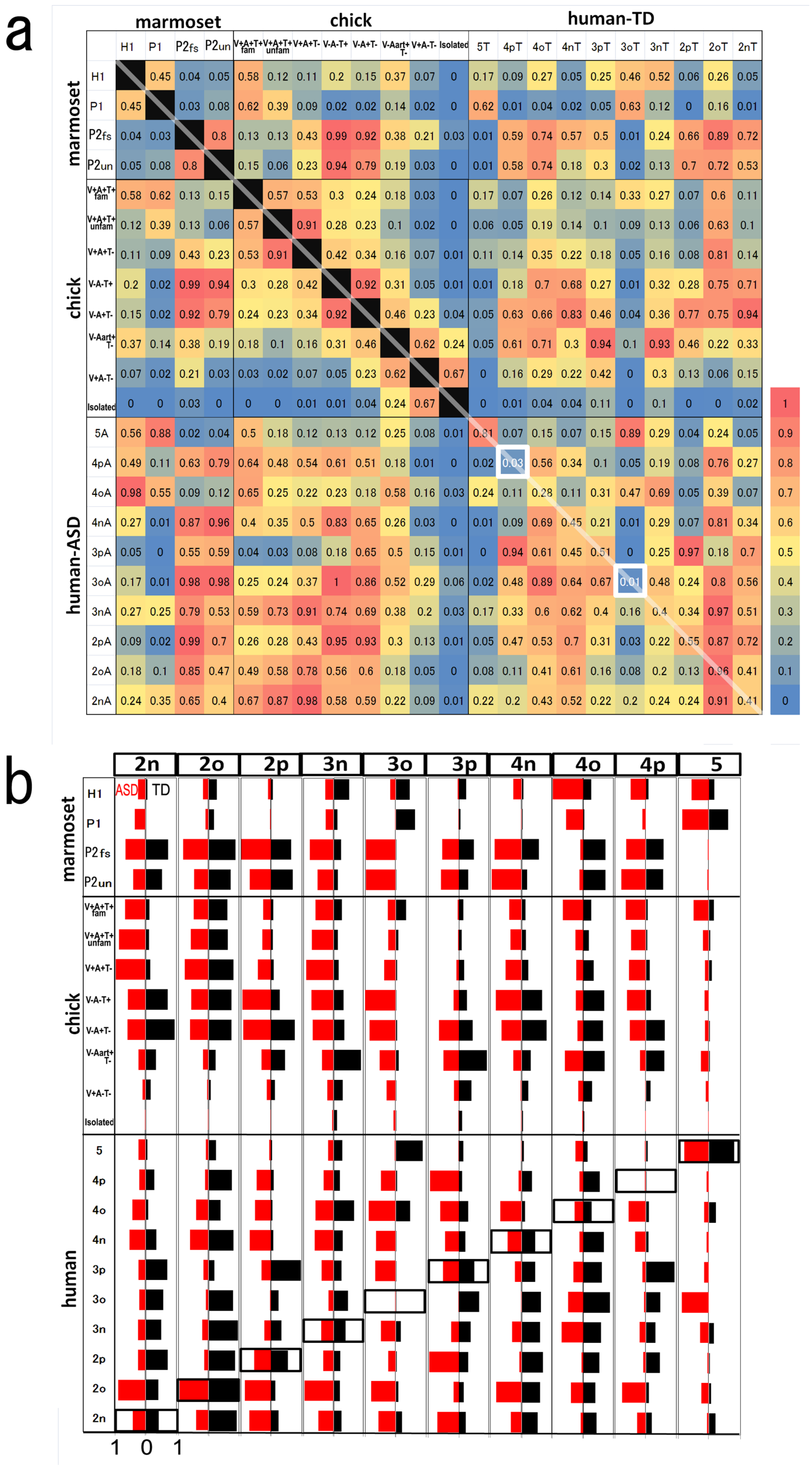
a
marmoset
chick
human-TD
H1 P1 P2fs P2un V+A+T+ fam V+A+T+ unfam V+A+T- V-A-T+ V-A+T- V-Aart+ T- V+A-T- Isolated 5T 4pT 4oT 4nT 3pT 3oT 3nT 2pT 2oT 2nT
H1 0.45 0.04 0.05 0.58 0.12 0.11 0.2 0.15 0.37 0.07 0 0.17 0.09 0.27 0.05 0.25 0.46 0.52 0.06 0.26 0.05
P1 0.45 0.03 0.08 0.62 0.39 0.09 0.02 0.02 0.14 0.02 0 0.62 0.01 0.04 0.02 0.05 0.63 0.12 0 0.16 0.01
P2fs 0.04 0.03 0.8 0.13 0.13 0.43 0.99 0.92 0.38 0.21 0.03 0.01 0.59 0.74 0.57 0.5 0.01 0.24 0.66 0.89 0.72
P2un 0.05 0.08 0.8 0.15 0.06 0.23 0.94 0.79 0.19 0.03 0 0.01 0.58 0.74 0.18 0.3 0.02 0.13 0.7 0.72 0.53
V+A+T+ fam 0.58 0.62 0.13 0.15 0.57 0.53 0.3 0.24 0.18 0.03 0 0.17 0.07 0.26 0.12 0.14 0.33 0.27 0.07 0.6 0.11
V+A+T+ unfam 0.12 0.39 0.13 0.06 0.57 0.91 0.28 0.23 0.1 0.02 0 0.06 0.05 0.19 0.14 0.1 0.09 0.13 0.06 0.63 0.1
V+A+T- 0.11 0.09 0.43 0.23 0.53 0.91 0.42 0.34 0.16 0.07 0.01 0.11 0.14 0.35 0.22 0.18 0.05 0.16 0.08 0.81 0.14
V-A-T+ 0.2 0.02 0.99 0.94 0.3 0.28 0.42 0.92 0.31 0.05 0.01 0.01 0.18 0.7 0.68 0.27 0.01 0.32 0.28 0.75 0.71
V-A+T- 0.15 0.02 0.92 0.79 0.24 0.23 0.34 0.92 0.46 0.23 0.04 0.05 0.63 0.66 0.83 0.46 0.04 0.36 0.77 0.75 0.94
V-Aart+ T- 0.37 0.14 0.38 0.19 0.18 0.1 0.16 0.31 0.46 0.62 0.24 0.05 0.61 0.71 0.3 0.94 0.1 0.93 0.46 0.22 0.33
V+A-T- 0.07 0.02 0.21 0.03 0.03 0.02 0.07 0.05 0.23 0.62 0.67 0 0.16 0.29 0.22 0.42 0 0.3 0.13 0.06 0.15
Isolated 0 0 0.03 0 0 0 0.01 0.01 0.04 0.24 0.67 0 0.01 0.04 0.04 0.11 0 0.1 0 0 0.02
human-ASD
5A 0.56 0.88 0.02 0.04 0.5 0.18 0.12 0.13 0.12 0.25 0.08 0.01 0.81 0.07 0.15 0.07 0.15 0.89 0.29 0.04 0.24 0.05
4pA 0.49 0.11 0.63 0.79 0.64 0.48 0.54 0.61 0.51 0.18 0.01 0 0.02 0.03 0.56 0.34 0.1 0.05 0.19 0.08 0.76 0.27
4oA 0.98 0.55 0.09 0.12 0.65 0.25 0.22 0.23 0.18 0.58 0.16 0.03 0.24 0.11 0.28 0.11 0.31 0.47 0.69 0.05 0.39 0.07
4nA 0.27 0.01 0.87 0.96 0.4 0.35 0.5 0.83 0.65 0.26 0.03 0 0.01 0.09 0.69 0.45 0.21 0.01 0.29 0.07 0.81 0.34
3pA 0.05 0 0.55 0.59 0.04 0.03 0.08 0.18 0.65 0.5 0.15 0.01 0 0.94 0.61 0.45 0.51 0 0.25 0.97 0.18 0.7
3oA 0.17 0.01 0.98 0.98 0.25 0.24 0.37 1 0.86 0.52 0.29 0.06 0.02 0.48 0.89 0.64 0.67 0.01 0.48 0.24 0.8 0.56
3nA 0.27 0.25 0.79 0.53 0.59 0.73 0.91 0.74 0.69 0.38 0.2 0.03 0.17 0.33 0.6 0.62 0.4 0.16 0.4 0.34 0.97 0.51
2pA 0.09 0.02 0.99 0.7 0.26 0.28 0.43 0.95 0.93 0.3 0.13 0.01 0.05 0.47 0.53 0.7 0.31 0.03 0.22 0.55 0.87 0.72
2oA 0.18 0.1 0.85 0.47 0.49 0.58 0.78 0.56 0.6 0.18 0.05 0 0.08 0.11 0.41 0.61 0.16 0.08 0.2 0.13 0.96 0.41
2nA 0.24 0.35 0.65 0.4 0.67 0.87 0.98 0.58 0.59 0.22 0.09 0.01 0.22 0.2 0.43 0.52 0.22 0.2 0.24 0.24 0.91 0.41
1 0.9 0.8 0.7 0.6 0.5 0.4 0.3 0.2 0.1 0
b
2n 2o 2p 3n 3o 3p 4n 4o 4p 5
ASD TD
marmoset
H1
P1
P2fs
P2un
chick
V+A+T+ fam
V+A+T+ unfam
V+A+T-
V-A-T+
V-A+T-
V-Aart+ T-
V+A-T-
Isolated
human
5
4p
4o
4n
3p
3o
3n
2p
2o
2n
1 0 1
